# Evaluation of three commercial rapid immunoassays for the diagnosis of *Clostridioides difficile* infection

**DOI:** 10.1128/spectrum.03405-24

**Published:** 2025-07-15

**Authors:** Hannes Bjarki Vigfússon, Theresa Ennefors, Torbjörn Norén, Martin Sundqvist

**Affiliations:** 1 Department of Laboratory Medicine, Clinical Microbiology, The Swedish Reference Laboratory for *Clostridioides difficile*, Örebro University Hospital 59566, Örebro, Sweden; 2 Faculty of Medicine and Health, Örebro University 6233, Örebro, Sweden; Johns Hopkins University, Baltimore, Maryland, USA

**Keywords:** *Clostridioides difficile*, diagnosis, rapid immunoassays, *Clostridioides difficile* infection

## Abstract

Current guidelines recommend the use of a two-step algorithm for the laboratory diagnosis of *Clostridioides difficile* infection (CDI). Several commercial rapid immunoassays that detect both GDH and toxin A/B in stool samples are available and could be used for both steps of the diagnostic algorithm. We aimed to evaluate the performance of three of these rapid immunoassays and study pathogen characteristics that might affect the sensitivity of toxin A/B detection. Leftover material from routinely collected stool samples from patients suspected of CDI was analyzed with the three immunoassays, nucleic acid amplification test (NAAT), and culture. Whole-genome sequencing was then used to evaluate *C. difficile* isolates recovered from the samples. The positive percent agreement between GDH detection and detection of *tcdA* by NAAT was 100% with all three immunoassays. The positive percent agreement for the detection of toxin A/B, however, was considerably lower (50.0%, 51.7%, and 71.4%), and freezing stool samples seemed to negatively affect the detection of both GDH and toxins. The isolates included in the study belonged to 23 different sequence types (ST) and all carried the *tcdA* and *tcdB* genes. The *C. diff* Quik Chek Complete performed the best of the three immunoassays, and when used in combination with NAAT, is a viable option for the laboratory diagnosis of CDI.

**IMPORTANCE:**

Laboratory diagnosis of *Clostridioides difficile* infection is complex, and current guidelines recommend a two-step diagnostic algorithm with a sensitive screening test and a more specific confirmatory test. The study aimed to evaluate three commercial rapid immunoassays that detect both glutamate dehydrogenase (GDH) and toxins A and B. These tests could be used for both steps of the two-step diagnostic algorithm and facilitate rapid laboratory confirmation of CDI, which is important for clinical decision-making and infection control measures.

## INTRODUCTION

*Clostridioides difficile* is an anaerobic, spore-forming, gram-positive rod that is common in the environment and can colonize the gastrointestinal tract of both animals and humans. *C. difficile* infection (CDI) causes antibiotic-associated diarrhea and pseudomembranous colitis and is one of the most common healthcare-associated infections worldwide (1–3). A wide variety of diagnostic tests are used to diagnose CDI, the most common being nucleic acid amplification tests (NAAT) that targets the *tcdA/tcdB* genes and enzyme immunoassays (EIA) that detect glutamate dehydrogenase (GDH) and free clostridial toxins (TcdA/TcdB) in stool samples (2, 4–8). NAATs are the most sensitive method but may lead to overdiagnosis, as asymptomatic carriage of *C. difficile* is common (4, 9, 10). Toxin EIAs are more specific for CDI, but their sensitivity is lower than that of NAAT (4). Detection of GDH by EIA has sensitivity similar to NAAT, but as GDH is produced by both toxigenic and non-toxigenic *C. difficile*, the test must be used in conjunction with another test that confirms the presence of toxigenic *C. difficile* (11). Reference tests like toxigenic culture (TC) and cell cytotoxicity neutralization assays (CCNA) are now seldom used for routine clinical diagnostics.

In 2016, the European Society of Clinical Microbiology and Infectious Diseases (ESCMID) updated its guidelines for the diagnosis of CDI and currently recommends a two-step diagnostic algorithm that combines a sensitive screening test (NAAT or GDH) and detection of free toxins with immunoassays (8). The Infectious Disease Society of America (IDSA) and the Society for Healthcare Epidemiology of America (SHEA) recommend a similar approach in their guidelines (7). This algorithm allows for optimal sensitivity but mitigates the risk of overdiagnosis by differentiating between probable (toxin positive) and possible (toxin negative) CDI (7, 8).

Several commercial rapid immunoassays that detect both GDH and toxins A and B are available (12–14). These assays generate rapid results and allow for the use of a single test for both steps of the recommended diagnostic algorithm. In this study, we evaluated the *C. diff* Quik Chek Complete (TechLab, Blacksburg, Virginia, USA), the CerTest *Clostridium difficile* GDH +Toxin A + B (CerTest, San Mateo de Gállego, Zaragoza, Spain), and the ImmunoCard STAT! *C. difficile* GDH-AB (Meridian Bioscience, Cincinnati, Ohio, USA). The diagnostic performance of the rapid immunoassays was compared to detection of the *tcdA* gene with the Alethia *C. difficile* (Meridian Bioscience, Cincinnati, Ohio, USA). We also performed a detailed characterization of the *C. difficile* isolates recovered in the study, using whole-genome sequencing (WGS), to identify pathogen characteristics that may affect the sensitivity of rapid immunoassays for the diagnosis of CDI.

## MATERIALS AND METHODS

### Sample collection

The study material consisted of stool samples sent to the Department of Laboratory Medicine at Örebro University Hospital in Sweden during the month of May 2019. The samples were collected from patients with suspected CDI as part of routine clinical practice. Only unformed stool samples were accepted for *C. difficile* testing, and samples were stored at 2**°**C–8**°**C for a maximum of 3 days before primary analysis. After routine diagnostic tests were done (NAAT and culture), the samples were anonymized and included in the study. The samples were then analyzed as soon as possible with the rapid immunoassays and cultured for the presence of *C. difficile*. All results were registered, and the *C. difficile* isolates were stored frozen at −70**°**C pending further analysis.

An additional 47 frozen stool samples, previously positive by NAAT, were also included in the study. The samples came from patients with suspected CDI and were collected as part of routine clinical practice during different periods of 2019. Routine analysis (NAAT and culture) was performed, after which the samples were anonymized and included in the study. The samples were stored frozen at −20**°**C and then thawed for analysis with the rapid immunoassays.

As all material that was used in the study was leftover material from clinical samples and anonymized, ethical approval from representative bodies was waived according to Swedish law. The study received all relevant institutional approval.

### Rapid immunoassays

The *C. diff* Quik Chek Complete is a membrane enzyme immunoassay that detects GDH and toxins A and B (a single result for toxin A/B) in stool samples (12, 13, 15). Analysis was performed according to the manufacturer’s instructions. Briefly, 25 µL of stool sample was diluted in 750 µL of dilution buffer and a drop of conjugate. After vortexing, 500 µL of the suspension was added to the test device, which was then incubated for 15 minutes at room temperature. After the incubation, 300 µL of wash buffer was added to the test device, followed by two drops of substrate. Results were read visually and recorded after 10 minutes.

The CerTest *Clostridium difficile* GDH +Toxin A + B is a chromatographic immunoassay that detects GDH and toxins A and B (separate results for toxin A and toxin B) in stool samples (16). Analysis was performed according to the manufacturer’s instructions. Briefly, 125 µL of stool was added to the stool collection tube containing diluent and homogenized by vortexing. Three drops of diluted sample were then added to each of the three sample windows on the test device. Results were read visually and recorded after 10 minutes.

The ImmunoCard STAT! *C. difficile* GDH-AB is a chromatographic immunoassay that detects GDH and toxins A and B (separate results for toxin A and toxin B) in stool samples. Analysis was performed according to the manufacturer’s instructions. Briefly, 110 µL of stool was added to the sample diluent vial and homogenized by vortexing. Four drops of the diluted sample were then added to each of the three sample windows. Results were read visually and recorded after 15 minutes.

In addition, all *C. difficile* isolates were analyzed with the *C. diff* Quik Chek Complete direct from colony to verify toxin production. One to three bacterial colonies, dependent on size, were added to the dilution buffer instead of stool material, but otherwise, the test procedure was the same as described above. Material for testing was collected from cultures on fastidious anaerobe agar after 48 hours of anaerobic incubation. The *C. diff* Quik Chek Complete assay is not approved by the manufacturer for testing bacterial colonies, and this was only performed for research purposes. Testing of bacterial colonies for toxin A/B should not be used in a clinical context unless appropriate validation studies have been done by the laboratory.

### Nucleic acid amplification testing

The Alethia *C. difficile* detects the *tcdA* gene in stool samples using loop-mediated amplification (LAMP) (17, 18). Analysis was performed according to the manufacturer’s instructions. Briefly, a small amount of stool sample was diluted in dilution buffer and heat-treated in a heat block at 95**°**C for 10 minutes. After the heat treatment, 50 µL of diluted sample material was transferred to a tube containing reaction buffer. After vortexing, 50 µL was then transferred to the test device and used in the LAMP reaction.

At the time of the study, the Alethia *C. difficile* was the routine method for laboratory diagnosis of CDI at Örebro University Hospital.

### Culture

Culture was done on all samples positive with NAAT and/or the rapid immunoassays. Selective agar was used to isolate *C. difficile* (ChromID *C. difficile*, Biomerieux, Marcy-l'Étoile, France). Plates were incubated under anaerobic conditions for 24–48 hours. MALDI-TOF MS was used to confirm species identification, and all *C. difficile* isolates were stored frozen at −70**°**C.

### WGS and multi-locus sequence typing

DNA extraction and sequencing using Illumina MiSeq (Illumina, San Diego, CA, United States) were performed as previously described by Werner et al (19). Multi-locus sequence typing (MLST) and core-genome MLST (cgMLST) were performed using the software Ridom SeqSphere+ (Ridom GmbH, Münster, Germany) and 1928D (1928 Diagnostics, Gothenburg, Sweden).

To compare the *tcdC* gene sequences of the study strains, the software BioEdit sequence alignment editor (Tom Hall, USA) was used to align the sequences to a wild-type *tcdC* sequence from reference strain ATCC 43255.

### Statistical analysis

Statistical analysis was done with GraphPad Prism version 10.5.1 for Windows (GraphPad Software, Boston, USA) and MedCalc Statistical Software for Windows version 20.218 (MedCalc Software bv, Ostend, Belgium). Positive and negative percent agreements were calculated with 95% confidence intervals. Cochran’s Q-test was used to assess differences in the sensitivity of the three immunoassays. A paired or unpaired *t*-test with a two-tailed p-value was used, as appropriate, to assess other differences. Statistical significance was defined as *P* < 0.05.

## RESULTS

### Consecutive stool samples

Fifty-seven consecutive stool samples from patients suspected of CDI were included in the study. All samples were analyzed with the Alethia *C. difficile* assay and the three rapid immunoassays. Results are presented in Table 1, and calculations of the diagnostic performance of the immunoassays compared to the Alethia *C. difficile* are shown in Table 2.

**TABLE 1:** Detection of GDH and toxins A/B with three rapid immunoassays compared to *tcdA* detection with the Alethia *C. difficile* (Meridian)

		Consecutive samples, *n* = 57	Frozen samples (positive with Alethia), *n* = 47
Alethia *C. difficile* (Meridian)		14	47
*C. diff* Quik Chek Complete (Techlab)	GDH	14	43
	Toxin A/B	10	26
*Clostridium difficile* GDH + Toxin A + B (Certest)	GDH	14/1^*a*^	42
	Toxin A/B	7/1*^a^*	18
Immunocard STAT! *C. difficile* GDH-AB (Meridian)	GDH	14	42
	Toxin A/B	8/1^*a*^	20

^*a*^ Positive samples that were negative with the study reference method (Alethia).

**TABLE 2:** Positive and negative percent agreement between three rapid immunoassays and the Alethia *C. difficile* on consecutive, fresh stool samples, % (95% CI)

		Positive % agreement	Negative % agreement
*C. diff* Quik Chek Complete (Techlab)	GDH	100 (76.8–100)	100 (91.8–100)
	Toxin A/B	71.4 (41.9–91.6)	100 (91.8–100)
*Clostridium difficile* GDH +Toxin A + B (Certest)	GDH	100 (76.8–100)	97.7 (87.7–99.9)
	Toxin A/B	50.0 (23.0–77.0)	97.7 (87.7–99.9)
Immunocard STAT! *C. difficile* GDH-AB (Meridian)	GDH	100 (76.8–100)	100 (91.8–100)
	Toxin A/B	51.7 (28.9–82.3)	97.7 (87.7–99.9)

One sample was positive with the CerTest assay (GDH+, toxin A+, and toxin B+) and the Meridian assay (GDH−, toxin A−, and toxin B+) but negative with the Techlab assay, Alethia *C. difficile* and selective culture. A single colony of *Clostridium hathawayi* was recovered from the sample, but there was no growth of *C. difficile*.

The positive percent agreement between GDH detection and NAAT was high for all three immunoassays, but considerably lower for toxin A/B detection. Of the three immunoassays, the *C. diff* Quik Chek Complete had the highest positive percent agreement. The difference, however, was not statistically significant, *P* = 0.472.

### Frozen stool samples

An additional 47 frozen stool samples were included in the study to further evaluate the sensitivity of the immunoassays. All frozen samples were positive for *tcdA* with the Alethia *C. difficile*. Results are presented in Table 1.

The positive percent agreement between GDH detection and NAAT in frozen stool samples was 91.5% (95% CI, 79.6−97.6), 89.4% (95% CI, 76.9−96.5), and 89.4% (95% CI, 76.9−96.5) for the *C. diff* Quik Chek Complete, *Clostridium difficile* GDH +Toxin A + B, and the Immunocard STAT! *C difficile,* respectively. The positive percent agreement between toxin A/B detection and NAAT in frozen samples was 55.3% (95% CI, 40.1−69.8), 38.3% (95% CI, 24.5−53.6), and 42.6% (95% CI, 28.3−57.8), respectively. The difference was statistically significant for toxin A/B (*P* = 0.006) but not for GDH (*P* = 0.779).

### Culture

Selective culture was done on all samples that were positive with the Alethia assay and/or with any of the rapid immunoassays. *C. difficile* was isolated from all 14 of the consecutive samples and 46 of 47 frozen samples, for a total of 60 isolates. The frozen sample that was negative by culture was positive for *tcdA* with the Alethia assay and positive for GDH but negative for toxin A/B with all three immunoassays.

As the *C. diff* Quik Chek Complete was considered the most sensitive assay based on the results from the fecal samples, all isolates were analyzed with this test directly from colony to verify toxin production. Fifty-seven (57) of 60 isolates were positive for both GDH and toxin A/B from the colony. Out of the three isolates that were positive for GDH but negative for toxin A/B, one isolate had been recovered from a stool sample that was positive for toxin A/B with the *C. diff* Quik Chek Complete but negative with the other two immunoassays. The other two isolates were from samples that were toxin A/B negative with all three immunoassays, except for a faintly positive toxin B result with the Immunocard STAT! *C. difficile* for one of the samples.

### Whole-genome sequencing

All isolates (*n* = 60) were whole-genome sequenced using Illumina sequencing. Multi-locus sequence typing showed that the isolates belonged to 23 different sequence types (ST), with ST2 being the most frequent (11 strains) followed by ST16 (6 strains). Only one of the strains belonged to ST1 (corresponding to ribotype 027). The *tcdA* and *tcdB* genes were present in all strains, and nine had the *cdtA/cdtB* genes (binary toxin) as well. The strain that belonged to ST1 had a single-nucleotide deletion at position 117 of the *tcdC* gene. Strains that belonged to ST5 (three strains) and ST11 (five strains) also had deletions in the middle of the *tcdC* gene. There was, however, no consistent trend regarding toxin A/B positivity in stool samples and *tcdC* deletions, as some of the strains were from toxin A/B positive samples while others were from negative samples.

## DISCUSSION

In this study, we compared three rapid immunoassays that detect GDH and toxins A and B with NAAT for the diagnosis of CDI. Like previous studies, we found GDH detection to be as sensitive as NAAT for the initial *C. difficile* screening, but the sensitivity of toxin detection was lower (12, 15, 20). Based on the consecutive samples, the *C. diff* Quik Chek Complete performed the best of the three immunoassays with a positive percent agreement of 71.4% for toxin A/B detection compared to NAAT, while the other two assays both had a positive percent agreement around 50%. This difference, however, was not statistically significant, likely because of the low number of samples in this arm of the study. For the frozen samples, we did see a statistically significant difference, with the *C. diff* Quik Chek Complete performing the best. Studies on the sensitivity of toxin detection by rapid immunoassays have generated mixed results, with some studies finding the sensitivity to be considerably lower than with well-type EIAs, while others have shown equal or even higher sensitivity (8, 12, 15, 20–23). The sensitivity we observed for the *C. diff* Quik Chek Complete is similar to that seen with well-type EIAs and is likely adequate, especially when reflex testing with NAAT is used for GDH-positive but toxin-negative samples (8). As the use of NAAT alone may lead to overdiagnosis of CDI by detecting carriers, it is reasonable that the sensitivity of toxin detection, which is believed to correlate better with clinical disease, is lower. If following the diagnostic algorithms recommended by ESCMID and IDSA/SHEA, positive samples can be reported as either probable CDI (GDH+, toxin A/B+) or possible CDI (GDH+, toxin A/B−, and NAAT+), hopefully mitigating the risk of overdiagnosis seen when NAAT is used alone (8).

One of the biggest benefits of rapid immunoassays is the fast turnaround time (TAT). Analysis takes less than 30 minutes, and as both double positive (GDH+, toxin A/B+) and double negative (GDH−, toxin A/B−) samples can be reported without further testing, most samples will have a very short TAT. Using the *C. diff* Quik Chek Complete as an example, 53 of 57 (93%) consecutive samples in the study would have been reported as either positive or negative without further testing, and only four (7%) would have had to undergo reflex testing with NAAT. As analysis is easy to perform and requires no specialized equipment, the need to centralize testing to specialized laboratories is avoided, making it possible to perform the analysis closer to the patient, thereby also reducing transport time.

All three immunoassays demonstrated a lower positive percent agreement for both GDH and toxin A/B detection in the frozen samples. For GDH, the positive percent agreement was around 10% lower, and for toxins A and B, around 10%–15% lower. This is troublesome, as all three manufacturers state that their tests can be used on frozen stool samples. They do note, however, that freezing and thawing cycles may affect sample quality. However, all the frozen samples in the study were promptly frozen within the timeframe stipulated by the manufacturers and only thawed for the analysis with the rapid immunoassays. Others have observed a similar difference in sensitivity between fresh and frozen samples for both GDH and toxins (14, 21, 24). Sharp et al. found that freezing and thawing samples had a negative effect on the sensitivity of GDH detection and speculated that this may explain the lower sensitivity of GDH compared to NAAT observed in some studies (14). This highlights an important limitation of immunoassays that both GDH and toxins are sensitive to degradation. This means that the pre-analytical handling of samples, as well as prompt analysis, is of importance for the quality of the results. We therefore advocate that frozen samples should not be used for routine clinical testing.

As previously mentioned, studies on the sensitivity of toxin detection have shown varying results. This might be related to the freezing and thawing of samples as discussed above; bias introduced by sample selection; or possibly some other factor related to the handling of samples that causes the toxins to degrade. Another possible explanation is antigenic variation of the toxins produced by different *C. difficile* strains. CDI is caused by a wide variety of *C. difficile* strains, and there are geographical differences in the epidemiology of strain types (25, 26). Local epidemiology may therefore affect the outcome of studies on toxin sensitivity, and the results may not be universally applicable. Because of the apparent hypervirulence and widespread occurrence of PCR ribotype 027 (RT027), many studies have been performed with material that heavily featured RT027. This can be a downside as some studies have shown RT027 to be easier to detect with toxin immunoassays than many other ribotypes, and most CDI in both the United States and Europe is caused by ribotypes other than RT027 (25–27). Our study included strains from 23 different sequence types, including one ST1 isolate (corresponding to RT027). All isolates were analyzed from the colony with the *C. diff* Quik Chek Complete, and all but three were positive for toxin, indicating that the test can detect toxin from a wide variety of STs. The three negative strains were from common sequence types (ST2, ST3, and ST54), all of which were represented by other toxin-positive isolates in the study material. There were too few isolates of each ST included to formally evaluate for correlation between specific STs and a positive or negative stool toxin test. There was, however, no obvious pattern seen.

The *tcdC* gene is a negative regulator of toxin A and/or B production in *C. difficile,* and loss-of-function mutations in this gene could theoretically lead to increased toxin production. Strains belonging to ST1/RT027 have a deletion at position 117 of the *tcdC* gene that leads to a frameshift and severe truncation of the gene (28, 29). This deletion is thought to be responsible for the hyperproduction of toxins and increased virulence of ST1/RT027 (29). Other deletions in the *tcdC* gene have been described but have not been conclusively shown to impact toxin production (30). We compared the *tcdC* genes of the isolates in the study and searched for deletions that could explain differences in toxin positivity. Only the isolate belonging to ST1/RT027 had the typical deletion at position 117. Several isolates had other deletions in the middle of the *tcdC* gene, but no associations between the deletions found in the *tcdC* gene and toxin positivity in stool samples were evident.

The study has some limitations. The number of consecutively collected samples was low, resulting in only 14 positive samples from that arm of the study. Frozen samples were used to evaluate positive percent agreement, but could not be used to evaluate negative percent agreement, as only NAAT-positive samples had been frozen. In retrospect, it would have been better to include only fresh samples and perform all the analysis at the same time, as freezing and thawing negatively affected the detection of both GDH and toxin A/B. We also deemed it necessary to exclude the frozen samples from some of the comparisons to get a fair assessment of the immunoassays. This likely affected the statistical calculations in a negative way. Lastly, as the study material consisted of anonymized samples, we were not able to gather clinical data and study the clinical sensitivity and specificity of the assays for predicting symptomatic disease.

The study also has several strengths, one being that we evaluated three different rapid immunoassays. The study was also done on routine clinical samples that reflect the local epidemiology and included many different STs. We also did a thorough characterization of the isolates recovered in the study with WGS.

In conclusion, we found rapid immunoassays that detect both GDH and toxins A and B to be a viable option for the diagnosis of CDI. The assays can be used for both steps of a two-step diagnostic algorithm as currently recommended by ESCMID and IDSA/SHEA. When used in combination with NAAT, they allow for both rapid and sensitive diagnosis of CDI. The detection of free toxins in stool also has the added benefit of a stronger clinical correlation than NAAT and allows for the stratification of positive samples into probable or possible CDI.

## Data Availability

The raw sequencing reads generated and analyzed during this study have been deposited in the NCBI Sequence read archive (SRA) under BioProject accession number: PRJNA1284618. The data are publicly available at: https://www.ncbi.nlm.nih.gov/bioproject/PRJNA1284618.
